# Effect of Microwave Application on the Xylan Extraction Yield from Agri-Waste Corncob for Sustainable Biomass Valorization: A Response Surface Methodology Optimization and Extract Characterization

**DOI:** 10.3390/molecules31183329

**Published:** 2026-09-19

**Authors:** Ayse Nur Bulut Gunes, Fatih Bozkurt, Nihan Sagcan, Osman Sagdic

**Affiliations:** 1 Department of Gastronomy and Culinary Arts, Faculty of Applied Sciences, Ozyegin University, Çekmekoy, 34794 Istanbul, Türkiye; 2 Department of Food Engineering, Faculty of Chemistry and Metallurgical Engineering, Yildiz Technical University, Esenler, 34220 Istanbul, Türkiye; fbozkurt@yildiz.edu.tr; 3 Department of Food Technologies, Corlu Vocational School, Tekirdag Namik Kemal University, Corlu, 59860 Tekirdag, Türkiye; nsagcan@nku.edu.tr

**Keywords:** xylan, extraction technologies, microwave, corncob, agri-waste, characterization, valorization of by-products

## Abstract

The global demand for sustainable resources highlights agricultural wastes for their valuable biomass content. Xylan, abundant in nature, offers biomedical applications in drug delivery, wound healing, and biodegradable food coatings. This study is the first in the literature to investigate the effect of microwaves on xylan extraction. The aim of this study is to investigate the effect of microwave application and determine the purity of the extraction outcomes in terms of xylan content. The microwave-assisted extractions were performed with two different diluted alkali solutions, potassium hydroxide (KOH) and sodium hydroxide (NaOH), and corncob was the biomass source for xylan. Experiments were designed with response surface methodology (RSM) to find the optimum conditions. Microwave energy demonstrated a statistically significant impact on extraction efficiency and yield (*p* < 0.05). Maximum yielding conditions for xylan extractions were found as follows: 1200-watt microwave power for 20 min with 10% (*w*/*v*) alkaline solution. The results of Fourier-transform infrared spectroscopy (FT-IR) and nuclear magnetic resonance (NMR) spectra of the KOH extract showed more resemblance to the xylan standard, with fewer indicators of impurities. However, the differential scanning calorimetry (DSC) and zeta potential analysis results of the two extracts did not differ from each other, showing similar thermal and suspension behavior. Xylan content of the KOH and NaOH extracts was determined as 77.82% and 72.01%, respectively.

## 1. Introduction

In 2018, the European Bioeconomy Strategy recognized agricultural and industrial wastes as sustainable sources for new valuable products [1]. Among these agricultural wastes, lignocellulose wastes are considered an ideal biomass feedstock for sustainability [2].

Xylan constitutes about 20–50% of lignocellulosic biomass in nature [3,4]. Xylan’s backbone consists of D-xylose units bound with β-(1–4)-links, which makes it indigestible [5]. Due to its indigestible nature, xylan has many positive effects on the human body, including supporting the immune defense system by promoting the growth of probiotic bacteria such as *Bifidobacterium* ssp. and *Lactobacillus* ssp., inhibiting cell mutation, and displaying anti-cancer activity [6,7,8,9,10].

Short-chain fatty acids produced by these probiotics support reductions in pH of the environment and increase the dissolution and absorption of minerals, such as calcium, magnesium, and iron. Absorption of these minerals promotes bone health and reduces the risk of osteoporosis [11]. Meanwhile, microbial fermentation results in the production of short-chain fatty acids, gases, and lactate, which may increase osmotic pressure and consequently increase fecal volume and intestinal motility [12]. Xylan is known as a prebiotic substance with numerous health benefits, particularly reducing the risk of cardiovascular diseases by lowering LDL cholesterol levels and reducing the risks of obesity and diabetes. As they accumulate in the intestines, they prevent the absorption of glucose into the blood and, thus, the rapid increase in the amount of insulin. They reduce serum cholesterol levels by ensuring the discharge and precipitation of bile acid in the intestine [13,14]. Since they cannot be broken down by enzymes in the mouth, they do not cause tooth decay and are, therefore, frequently used in industry [15]. Xylan has also gained attention in different industries, such as packaging- and chemistry-oriented industries. Due to its biodegradability, xylan has attracted considerable attention as a potential material for bioplastic applications [16] and drug delivery systems [17]. All these findings continuously increase the interest in xylan. Sources of xylan can vary since it is one of the major components of lignocellulose biomass [18,19]. Hardwood materials and relatively softer materials, such as sunflower stalks, cotton stalks, tobacco stalks, corncobs, sugarcane bagasse, banana fibers, wheat straw, and paddy and barley husks [20,21], can be the objectives of xylan extraction.

Xylan extraction can be classified according to the extracting agent, such as alkaline, acidic, enzymatic, and, in the case of water, autohydrolysis. Autohydrolysis is a single-step extraction using high temperatures. It was reported that autohydrolysis and acid hydrolysis methods yield xylan in relatively lower yields and create impurities such as water-soluble lignin, many monosaccharides and their degradation components, making them less advantageous options of extraction [22]. Even though high temperatures increase the extraction yield by breaking the bonds between the lignin and xylan chain [23], they may also cause hydroxymethylfurfural (HMF) formation, which can inhibit the prebiotic effect [24] with other impurities. On the other hand, acidic treatments produce large amounts of xylose and toxic compounds while causing corrosion on the equipment and raising environmental concerns [25]. These reasons make dilute alkaline treatment a better option for food-grade productions [26]. The studies comparing the extraction methods reported the dilute alkali extraction as the highest-yielding method [27]. Alkaline reagents at mild concentrations have been shown to break the lignin–cellulose and hemicellulose–cellulose hydrogen bonds as well as the ester bond between lignin and hemicelluloses, leading to efficient xylan solubilization [28]. Based on its solubility characteristics, corncob xylan is classified into two forms: water-soluble and water-insoluble (the latter known as the prebiotic form) [29].

Apart from the agents of hemicellulose extraction, the techniques can be another point for categorization such as ultra sonification and screw extrusion [30].

Extractions can be improved by combining different technologies to increase the yield, shorten the extraction time or decrease the solvent or energy usage. One of these technologies is microwave application. In microwave-assisted extraction, polar molecules are selectively heated by the microwave energy. The electric field of microwaves causes heating via dipolar rotation and ionic conduction [31]. Ionic conduction is the electrophoretic migration of ions under electromagnetic field application. The resistance of the solution to this flow creates friction and this heats the solution. The second mechanism, dipole rotation, is the realignment of dipoles with the applied field that happens 4.9 × 10^9^ times per second, and this movement heats the medium. Dipole rotation also breaks weak hydrogen bounds, which enhances the extraction. With the migration of dissolved ions, solvent penetration into the matrix increases [32]. Using closed vessels for microwave application, the extraction can be performed at elevated temperatures for shorter durations of 15–30 min and with fewer volumes around 10–30 mL. In addition, many samples can be extracted at the same time. On the other hand, a reduction in energy and time also decreases the cost. Improved yield and product uniformity result in higher-quality extracts compared to other extraction techniques [33].

This study represents the first report to investigate the microwave effect of xylan extraction. In this study, we conducted systematic optimization of microwave-assisted alkaline extraction of xylan from corncobs with the aim of increasing the extraction yield while decreasing the extraction time and amount of alkaline. Two types of alkaline, sodium hydroxide and potassium hydroxide, at different concentrations under different microwave lengths and durations were examined. Response surface methodology (RSM) was used to investigate the optimum conditions for the closed-vessel microwave-assisted xylan extraction from corncobs using dilute alkaline solutions. Box–Behnken Design, consisting of three factors, microwave power, duration and concentration of the dilute alkaline, was used. Furthermore, the samples of optimized extract mass yield conditions were tested for xylan content with high-performance liquid chromatography (HPLC), and characterization analyses were performed with Fourier-transform infrared spectroscopy (FT-IR), nuclear magnetic resonance (NMR) spectrometry, differential scanning calorimetry (DSC) and zeta potential analysis.

## 2. Results and Discussion

### 2.1. Xylan Extraction

The results of microwave-assisted extraction are given in Table 1 as the percentage mass of extract relative to the mass of the corncob. Extractions with NaOH (Na-X) resulted in higher mass yield compared to the extractions with KOH (K-X). Increased alkali concentrations resulted in higher mass yield. The highest yield is obtained in Na-X with 10% concentration assisted with 800-watt microwave for 20 min. In a study where xylan was enzyme aid-extracted from barley husk through pre-treatments over long hours that took more than two days, the yield was found to be 40% (*w*/*v*) [34]. Meanwhile, our microwave-assisted extraction process took less than 3 h to obtain 43.74% from corncob. In another study, xylan was extracted from corncob with deep eutectic solvent at 60 °C for 3 h and produced a 16.46% yield [35]. In our microwave-assisted extraction, we were able to obtain 31.49% of the biomass as xylan in 20 min of extraction. Microwave application increased the yield of xylan extraction from corncob while shortening the process duration.

Through RSM (response surface methodology) analysis, the optimum conditions for the microwave-assisted extraction were found to be 1200 W of microwave power application for 20 min with 10% of alkali solution. The results showed that the increased alkali solution concentration increased the extraction mass yield. This finding aligns with results of studies where xylan extraction was investigated [21,27]. We obtained higher mass yield with NaOH extractions, which is in alignment with a study where steam-assisted xylan extraction from sugarcane bagasse with sodium hydroxide and potassium hydroxide was compared, and sodium hydroxide was also found to be superior to potassium hydroxide for achieving higher recovery of xylan [36]. However, increased concentration of alkaline results in an increased amount of salts in the neutralization step, which is a common problem for all alkaline extractions [37]. The highest-yielding KOH extraction was 10% KOH solution, with 1200-watt microwave for 11 min. The lowest mass yield was obtained with both alkaline solutions with 1% solution concentration.

In the final model presented in Table 2, non-significant terms (*p* > 0.05) were omitted by backward elimination after the first run of ANOVA (analysis of variance) to focus on factors with significant main and interaction effects. The terms that are not significant for one of the alkalis is represented as NS (non-significant). The reduced model for K-X incorporated first-order terms and two-way interaction of microwave power and alkali concentration (AC, *p* = 0.02) with insignificant lack of fit (*p* = 0.275) and an R^2^ = 81.43% (Table 3) value, confirming the validity of the model. Extraction time did not show a significant effect with KOH.

On the other hand, the Na-X model represented linear parameters, time and alkali concentration interaction (BC, *p* = 0.003) and a full second-order polynomial (quadratic) structure featuring significant curvature for alkali concentration (C^2^, *p* = 0.001) with R^2^ = 95.57% (Table 3).

Empirical regression equations derived from the optimized models are given below:

(1) $${\mathrm{Extraction}\;\mathrm{Yield}}_{K\text{-}X}\left(\%\right)=7.14-0.008\left(A\right)-0.84\left(C\right)+0.004\left(AC\right)$$

(2) $${\mathrm{Extraction}\;\mathrm{Yield}}_{Na\text{-}X}\left(\%\right)=-5.64+0.0096\left(A\right)-0.174\left(B\right)+4.67\left(C\right)-0.3487\left(C^{2}\right)+0.150\left(BC\right)$$

**Figure 1:**
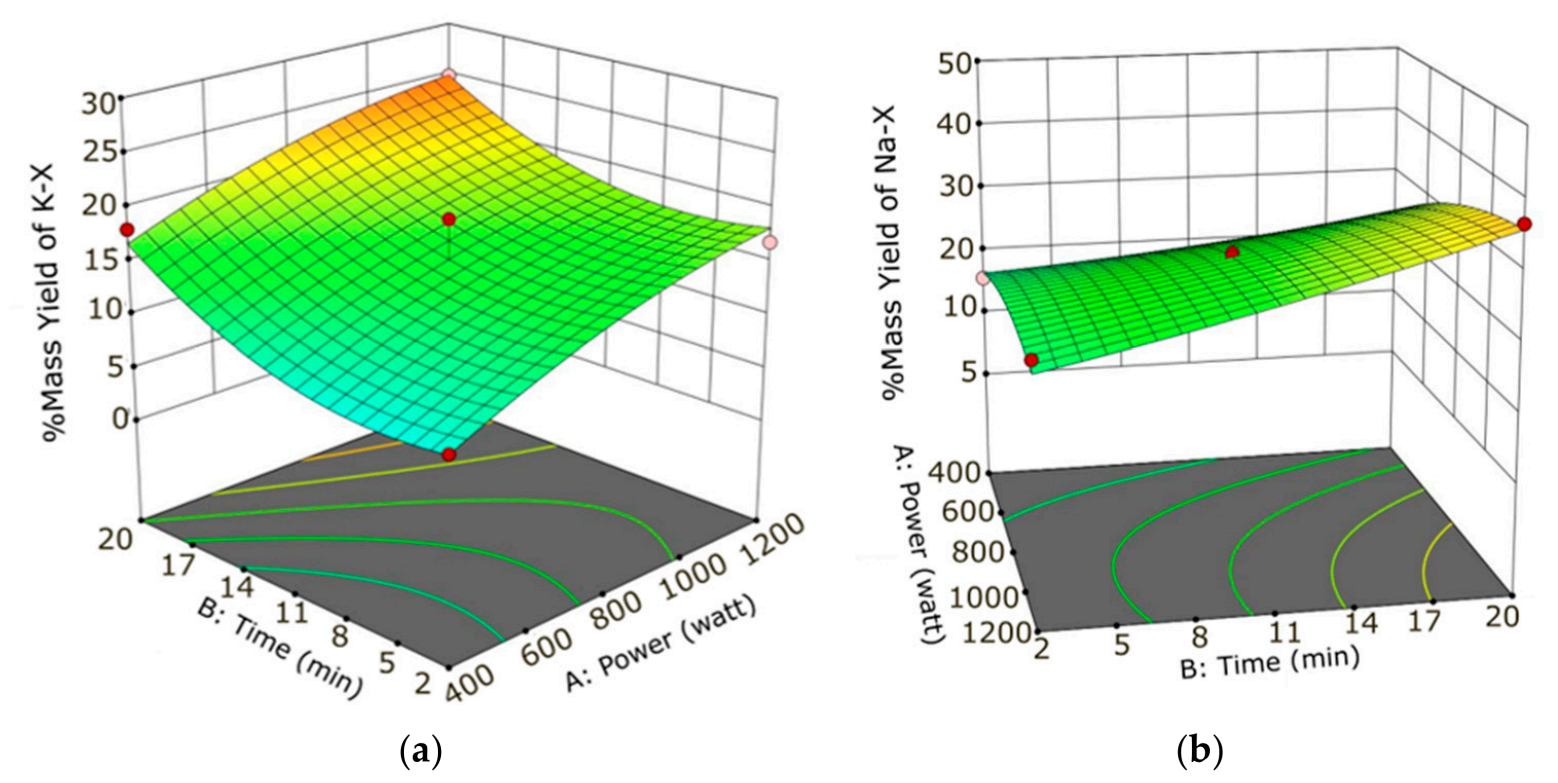
3D Response surface plot of (**a**) K-X (extraction with KOH) and (**b**) Na-X (extraction with NaOH). Red dots: above the surface, pink dots: below the surface.

Figure 1a,b show the 3D surface response plots of K-X and Na-X, respectively, where the concentration of the alkali solution was fixed to 5.5% (center value of the testing range), with axis A being microwave power (Factor A) in watts and axis B extraction time (Factor B) in minutes. The red dots represent the experimental results that are above the RSM model, and pink dots represent the experimental results that are below the model. Both dots being located close to the surface indicates a good fit of the model to the experimental results. In Figure 1a, the curve on the surface and the elliptical shape of the contour plots show a synergistic interaction between watts (Factor A) and time (Factor B).

The steep slope on axis B (time) emphasizes the effect of the time on the yield of Na-X in Figure 1b. Also, the steeper curve of the counter lines at the bottom of the graph suggests a stronger statistical interaction between time and power in Na-X (Figure 1b) than K-X (Figure 1a). Even though the increasing microwave power and time have positive effects on the response, the curve of the plot on Axis A in Figure 1a suggests a further increase in power or time of the microwave application might decrease the yield. This may be due to the degrading effect of irradiation or the alkaline.

**Figure 2:**
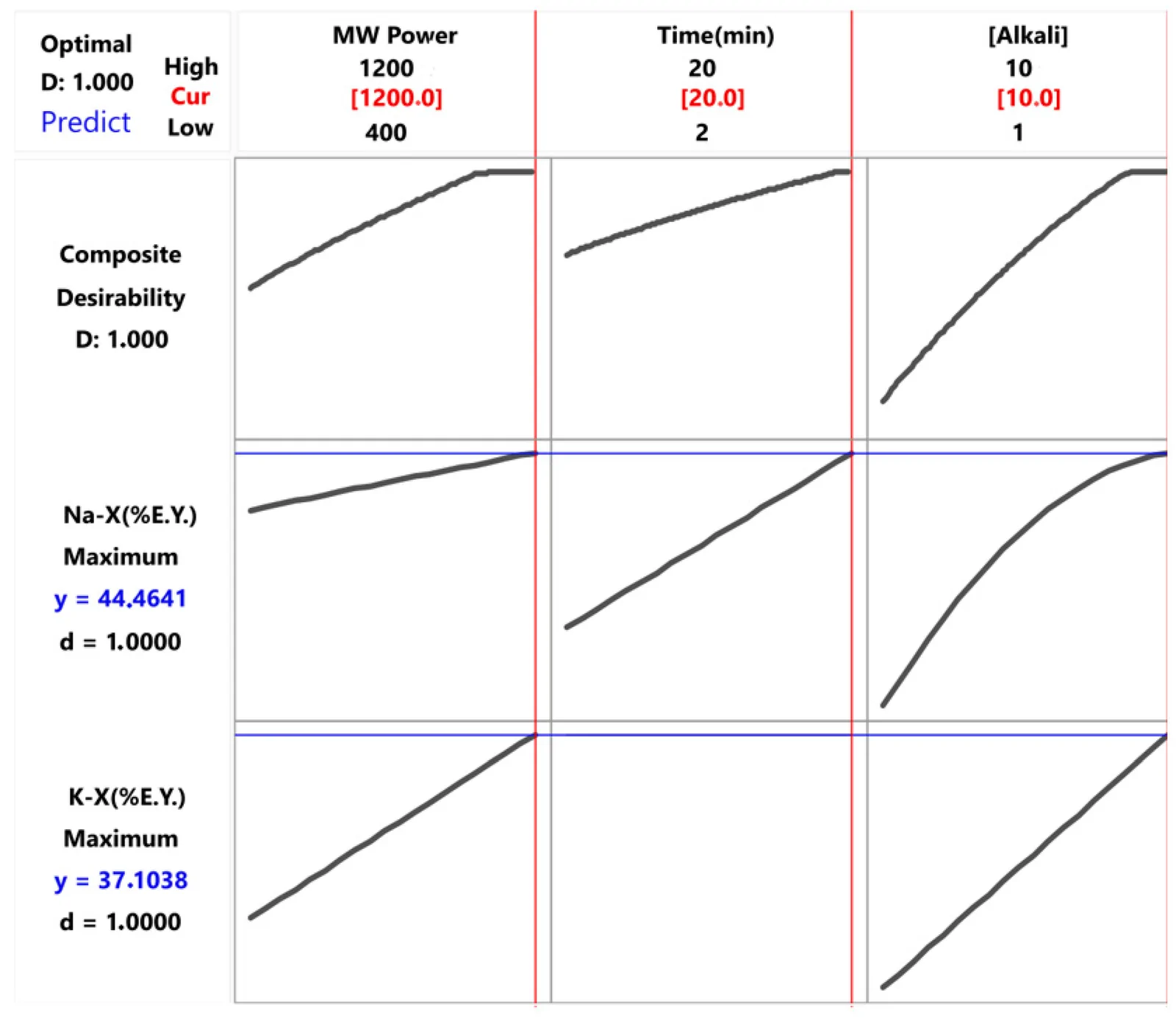
Response optimization of factors.

Process optimizations to maximize the extract yield % were conducted independently for K-X and Na-X. Both optimization procedures achieved maximum desirability (*d* = 1.000) within the experimental space (Figure 2). An increase in all three independent factors affected the yield positively. Optimum conditions for the maximum yield were found to be the maximum limits within the experimental design, which are 1200-watt microwave power for 20 min with 10% alkali concentration. For the optimum model of Na-X, the alkali concentration formed a curve as an onset of a parabolic trend. This curve indicates that an increase in alkali concentration may result in a marginal decrease in extraction yield, which may be a result of the further structural degradation of xylan as an effect of microwaves. However, determining the absolute optimum conditions for maximum extraction yield requires further investigation as responses showed an increase within the experimental design range, and none reached a vertex or plateau.

In a previous study investigating ultrasound waves’ effect on xylan extraction from corncobs, the short ultrasound treatment (up to 30 min) was found to compound the extraction process of the water-soluble xylan [38]. Although time was eliminated during the optimization of K-X, 20 min was preferred as the duration in accordance with the selected process conditions. Characterization analysis was performed with the optimized extract. Experiments conducted under optimized conditions resulted in 43.78% and 34.21% for Na-X and K-X, respectively. These values correspond to a relative deviation of 1.29% for Na-X and 7.8% for K-X, both within the prediction interval.

### 2.2. Extract Characterization

#### 2.2.1. DSC

**Figure 3:**
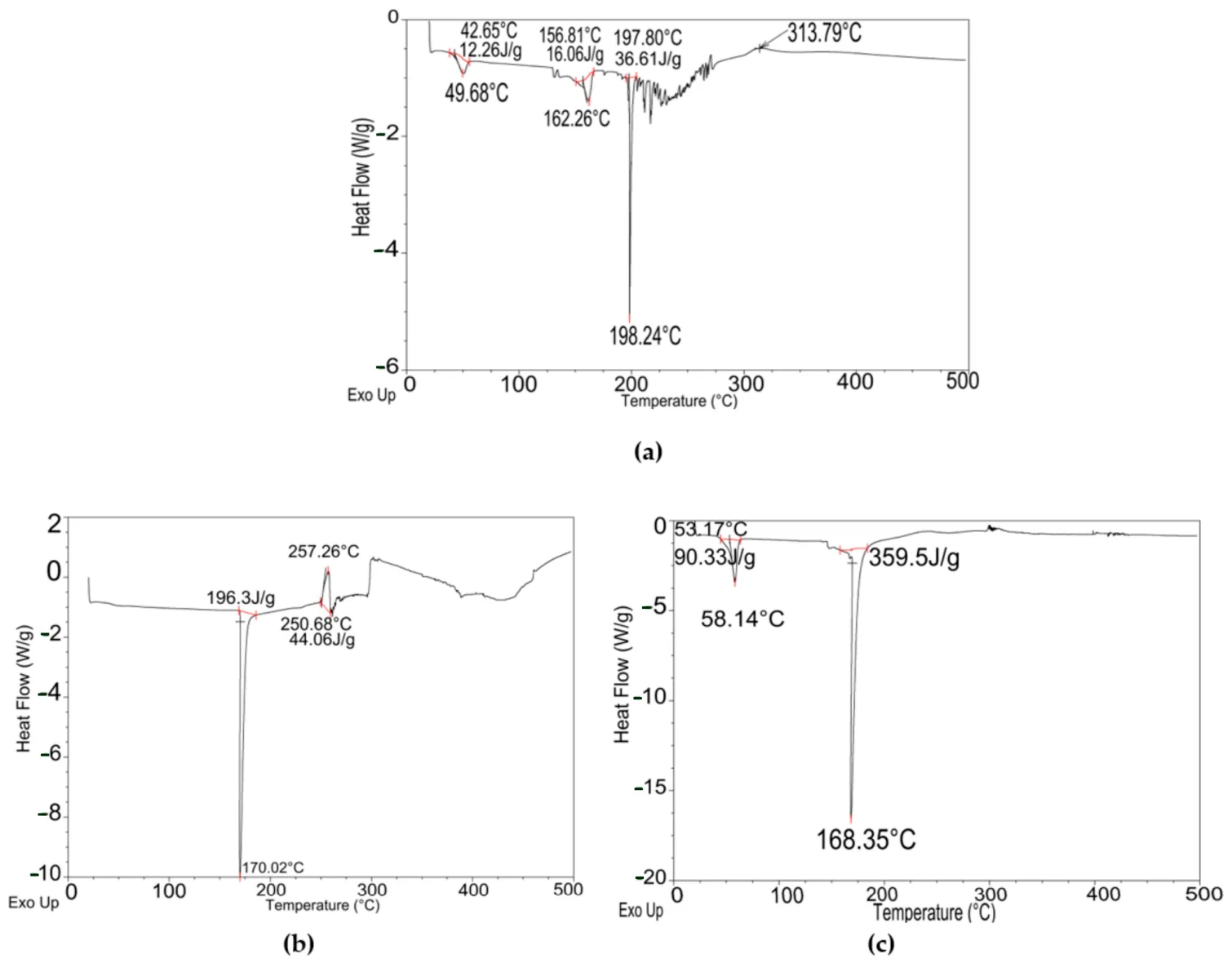
DSC plots of the (**a**) xylan standard; (**b**) K-X (extraction with KOH); (**c**) Na-X (extraction with NaOH).

DSC (differential scanning calorimetry) was performed to see the thermal behavior of the extracts compared to the standard. The plots of DSC analysis of the xylan standard (a) K-X (b) and Na-X (c) are given in Figure 3. The major decomposition temperature of the xylan standard was 198.24 °C (Figure 3a) with an enthalpy of 36.61 J/g, corresponding to the thermal degradation of the polysaccharide backbone. A further decomposition of carbonaceous residues was at 313.79 °C. These results are in correlation with thermal profiles of xylan reported in the literature, where the onset of thermal degradation typically begins around 150 °C, and it keeps losing mass up to 243 °C [39,40]. Similar degradation peaks were also found at 170.02 °C with 196.3 J/g enthalpy (Figure 3b). The lower decomposition temperatures of the alkaline extracts compared to the standard may be attributed to the low xylan concentration in the extracts and/or impurities present in them [40].

#### 2.2.2. Zeta Potential

Zeta potential analysis was conducted to see the surface charge, colloidal stability and the effect of the residual ions on the surface of the extract, compared to the commercial standard. The mV results of zeta potential are given in Table 4. The xylan standard had the highest negative zeta potential value, meaning a higher degree of surface charge and electrostatic stability in aqueous dispersion, since higher absolute values of zeta potential indicate more polar stability in solutions, and as the absolute value decreases, phase separations may increase [41]. Even though zeta potential values between 0 and ±10 are categorized as highly unstable [42], they may not provide the whole picture of colloidal stability [43]. All the charges were found to be negative in our study, which aligns with the zeta potential charges of xylan from beech and spruce that were found to be negative, and as the pH of the medium increased, zeta potential of the xylan decreased; however, phase separation was not observed [44]. In another study, where chitosan was added to the xylan structure, the suspension became cationic [45], which may be related to the excess amine groups of chitosan [46]. The absolute zeta potential values of Na-X and K-X were found to be lower than the standard; however, alkali cations did not show a significant difference between each other.

#### 2.2.3. FT-IR

**Figure 4:**
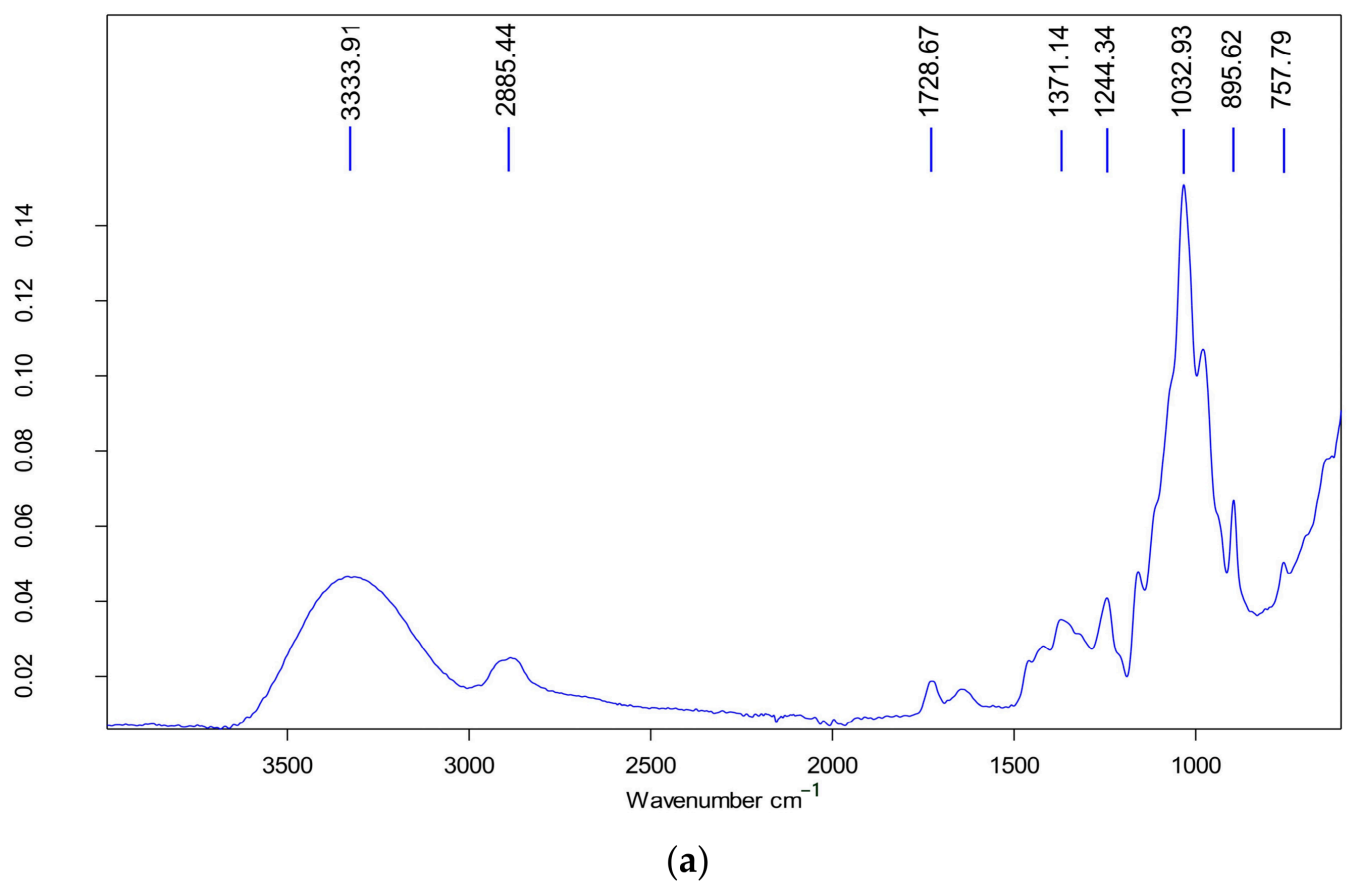

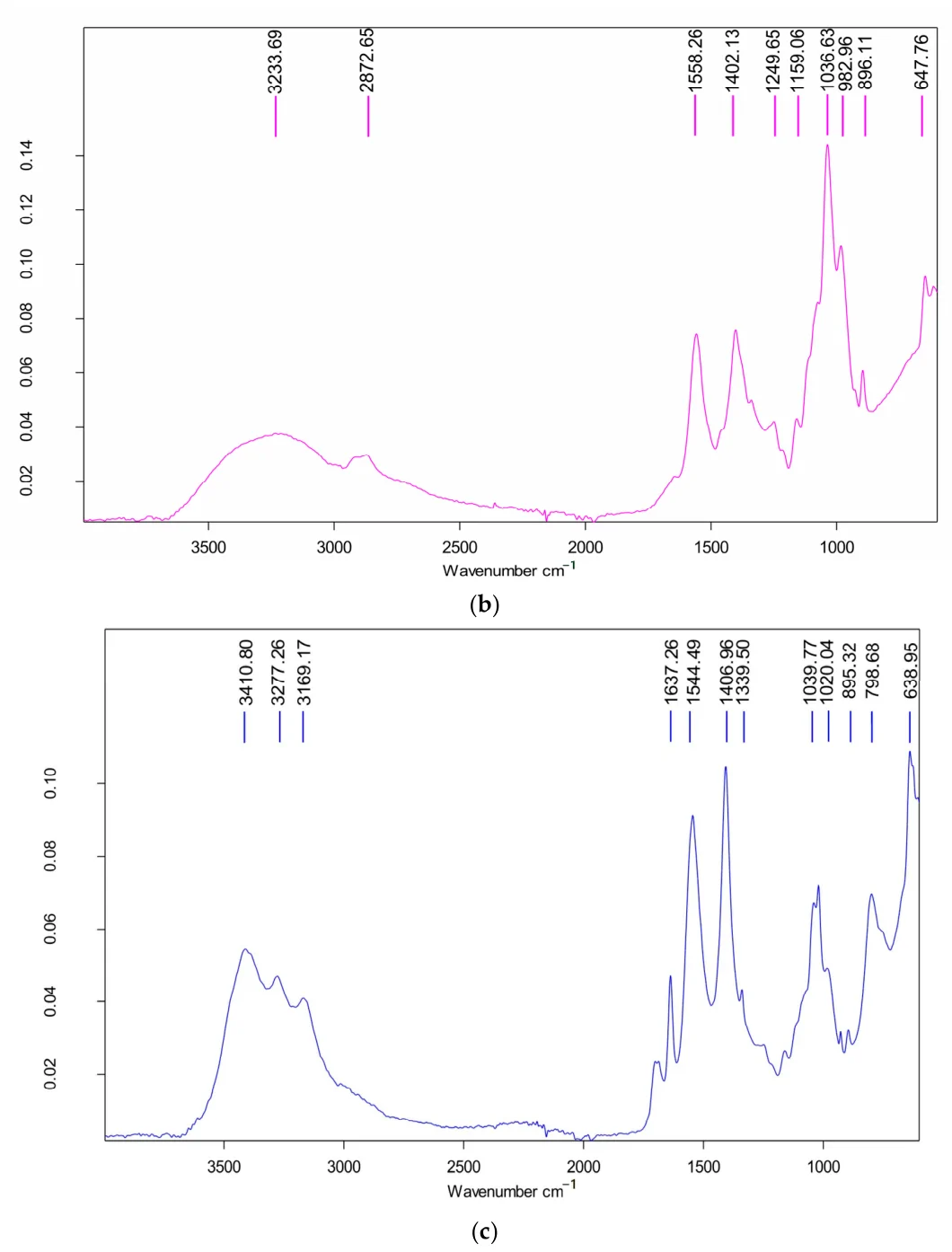
FT-IR plots of the (**a**) xylan standard; (**b**) K-X (extraction with KOH); (**c**) Na-X (extraction with NaOH).

FT-IR (Fourier-transform infrared spectroscopy) is a fast option to investigate the physicochemical and conformational properties of carbohydrates [47]. It can also be used to see the purity of the samples through absorption band patterns [48]. We aimed to compare the FT-IR spectra of extraction samples against the spectra of the corncob xylan standard. Graphs of FT-IR spectra of the xylan standard (a) and extractions with KOH (b) and NaOH (c) are given in Figure 4. The absorbance peaks of the standard and K-X in the analytical region, especially between 3500 and 2500 cm^−1^ wavelengths, were very similar, which refers to carboxylic acids. The peak at 3300 cm^−1^ is an indicator of -OH stretching vibration, showing all the samples had hydroxyl groups [49]. All the spectra showed similar patterns between 2000 and 2500 cm^−1^ wavelength. The wavelength region below 1500 cm^−1^ is known as the fingerprint, and samples of K-X showed greater resemblance to the standard. The peaks around 1200–1000 cm^−1^ are known to be specific to xylan and were present in all of the spectra [50]. The absorbance around 1030 and 895 cm^−1^ was present in all of the spectra. It was also stated in a study investigating xylan extraction from sugarcane bagasse that the absorbances at 3438, 1414, 1033 and 808 cm^−1^ were associated with xylan [36]. In another study, the peak at 850–890 at cm^−1^ was thought to be the β (1–4)-glycosidic link in the xylan structure amongst the xylose units [51].

#### 2.2.4. Xylan Content and Degree of Polymerization

To estimate the xylose content, samples and the xylan standard were treated with H_2_SO_4_, as described below. The calibration curve of the xylose standard from HPLC (high-performance liquid chromatography) was obtained with R^2^ = 0.987, and the curve of the spectrophotometer was obtained with R^2^ = 0.9994. Obtained xylose concentrations were used to estimate both the xylan content and the average polymerization degree (avDP) of the samples.

A correction factor of 0.88 is used to determine the xylan content, and the results are given in Table 5 as the % (*w*/*w*) of the xylan mass to the total mass of the extract.

The results revealed that with NaOH solution, 33.8% of the biomass, and with KOH solution, 24.8% of the biomass were extracted as xylan. As xylan makes up between 25 and 39% of the corncob, obtained yields indicate substantial recovery of the xylan fraction [52,53,54]. The xylose content of the extracts was found to be 68.2 and 74.6% for Na-X and K-X, respectively, which aligns with the 70 and 66.4% results of previous studies [55,56]. While high-DP xylan is required in material applications, low-DP xylan may be preferred for health-promoting prebiotic productions.

#### 2.2.5. NMR

**Figure 5:**
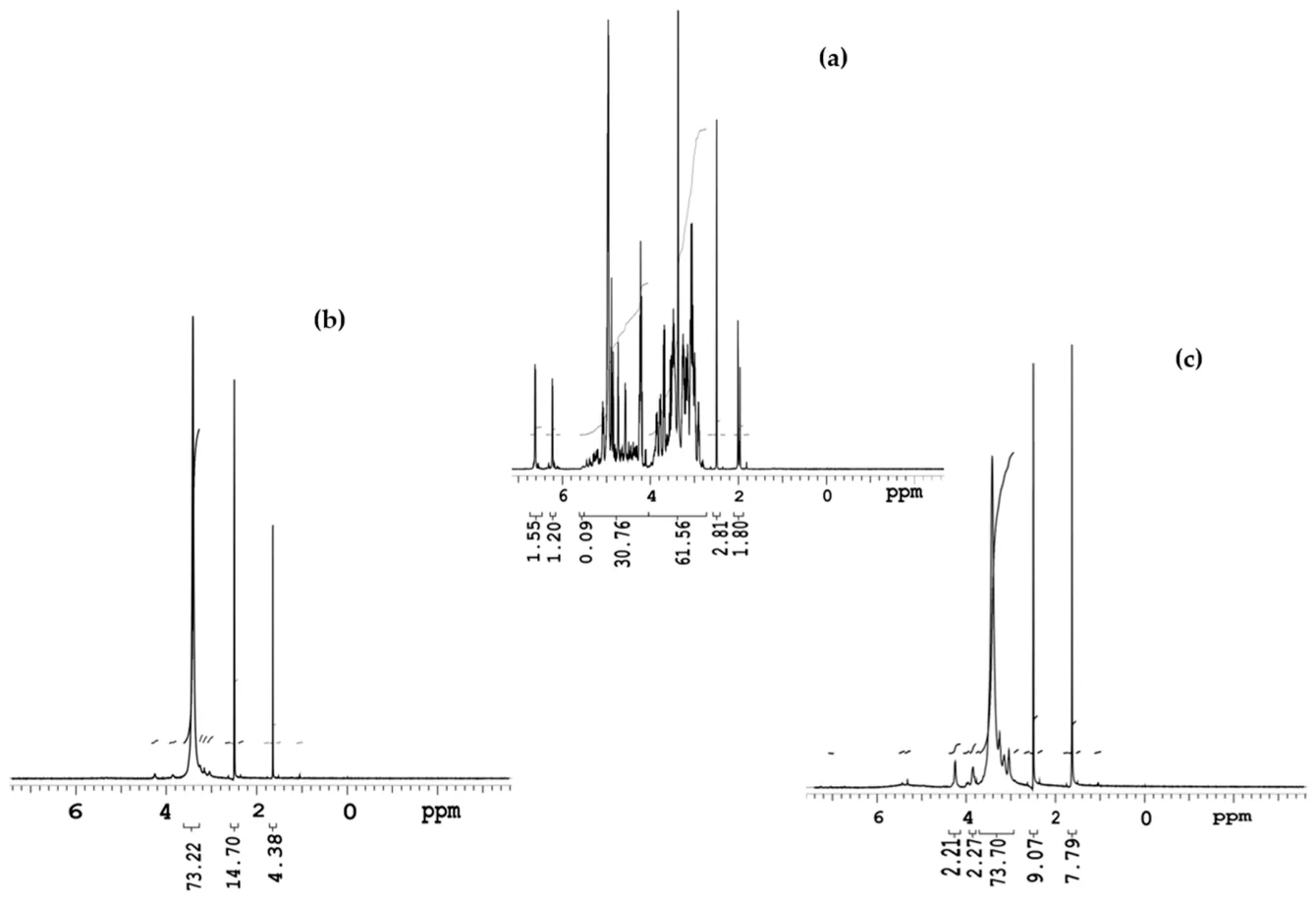
NMR spectrum of (**a**) xylan standard, (**b**) K-X (extraction with KOH), (**c**) Na-X (extraction with NaOH).

The ^1^H NMR (nuclear magnetic resonance) spectrum of the xylan standard (Figure 5a) exhibited typical signals for polysaccharides, with dense peaks between 3.0 and 5.5 ppm. The signals between 3.0 and 4.4 ppm are accredited to D-xylose [57].

Both extracts displayed similar polysaccharide signals in their NMR spectra; however, additional peaks were observed in the aliphatic (0.5–2.5 ppm) and aromatic (6.0–8.0 ppm) regions. In a study in which xylan from corncob was extracted and purified, samples did not show any peaks in the aliphatic regions, similar to our xylan standard [58]. The sample K-X had fewer impurity signals (Figure 5b) compared to Na-X (Figure 5c). The spectra between 3 and 4.3 ppm are an indicator of xylose presence in a xylan extract from corncob, which is also visible both in Na-X and K-X samples [59].

## 3. Materials and Methods

### 3.1. Sample Preparation

All the chemicals used in this study are analytical grade and from Sigma-Aldrich (Merck KGaA, Darmstadt, Germany). Xylan from corn cob is from Tokyo Chemical Industry Co., Ltd. (TCI, Tokyo, Japan).

Ground corncob was obtained from a local producer (Akin Tarim Urunleri, Sakarya, Turkey) of 0.6–0.75 mm particle size. The ground corncobs were ground to finer dust with a seed processor (Sinbo, Istanbul, Turkey). Finely ground corncob dust was pre-treated in Soxhlet extractor (Wiggens GmbH, Wuppertal, Germany) for 24 h with acetone and ethanol, respectively, to remove lipids and impurities to improve the extraction. Pre-treated corncob dust was dried at 65 °C for 3 h in a forced-air oven (UF450, Memmert GmbH + Co. KG, Schwabach, Germany).

### 3.2. Xylan Extraction

Here, 5 g of pre-treaded corncob was added to 80 mL of alkali solution and stirred for 5 min on a magnetic stirrer (Labnet International, Edison, NJ, USA) to ensure complete soaking of the samples. Samples were placed in a microwave extractor (Milestone Inc., Sorisole, Italy). Two different alkali solutions, NaOH and KOH, were chosen for the extraction. Concentration of the samples, duration and the power of the microwave to be applied were determined with RSM. The experimental design was formed with RSM based on Box–Behnken Design using three factors, microwave power, duration and alkaline solution concentration, to find out the maximum-yielding conditions for xylan extraction by using Minitab version 17 software. The independent variables and treatment codes are given in Table 6.

The running software resulted in 15 treatments with independent variables as watt, time and concentration, and the response as the yield. The experimental design can be seen in Table 7. The extractions in microwave extractor took place at different power levels and times according to the design for each alkaline solution. Extraction mass yield was calculated as Equation (3):

(3) $$Yield\;(\%)=\frac{mass\;of\;freeze\;dried\;sample\;(g)}{mass\;of\;the\;corncob\;(g)}\times 100$$

After the microwave treatment, samples were filtered after centrifuge (Nuve, Ankara, Turkey) at 4000 rpm for 8 min, and pH of the filtrate was fixed to 4.8 on a magnetic stirrer with 1M acetic acid with the help of a pH meter (Mettler-Toledo GmbH, Greifensee, Switzerland). Twice the volume of the solution of ethanol was added to the mixture and centrifuged in a high-speed refrigerated centrifuge (Hitachi, Tokyo, Japan) at 1000 rpm for 5 min at 4 °C. The precipitate was obtained and frozen at −80 °C in a deep freezer (Sanyo Electric Co., Ltd., Osaka, Japan) until freeze-drying for 72 h in a freeze dryer (Martin Christ, Osterode am Harz, Germany).

Severity of the microwave applications on the sample is calculated as the amount of energy per mass, as given in Equation (4).

(4) $$Severity\;(J/g)=\frac{Energy\;(Power\;(watt)\times time\;(s))}{sample\;mass\;(g)}$$

### 3.3. Methods of Characterization

Xylan standard and the samples with the maximum yield from both alkaline solutions were tested for characterization with FT-IR (Fourier-transform infrared spectroscopy), DSC (differential scanning calorimetry), zeta potential analysis, HPLC (high-performance liquid chromatography), and NMR (nuclear magnetic resonance) spectrometer.

#### 3.3.1. DSC

For DSC analysis, 5 mg of sample was placed into a pan. The samples were heated from 20 °C to 500 °C, with 10 °C/min rate in DSC instrument with nitrogen (DSC Q20, TA Instruments, New Castle, DE, USA) [40].

#### 3.3.2. Zeta Potential

Zeta potential was analyzed with Zetasizer Nano (Malvern, Worcestershire, United Kingdom), and 5 mg of samples was prepared in phosphate-buffered saline solution and loaded in disposable folded capillary tubes [46]. The prepared solutions were measured three times per sample.

#### 3.3.3. FT-IR

FT-IR analysis was performed with a Bruker Tensor 27-FT-IR Spectrometer (Billerica, MA, USA) equipped with a DLa TGS detector (Bremen, Germany). Thus, 5 mg of freeze-dried samples was analyzed at room temperature in a range of 4000–400 cm^−1^ wavelength using 16 scans at a resolution of 4 cm^−1^ at intervals of 1 cm^−1^ [36].

#### 3.3.4. Xylan Content and Degree of Polymerization

In order to estimate the polymerization degree of the extracts, xylose and reducing sugar contents were determined and compared to the standard xylan. Since each chain has one reducing end, dividing the total quantity of xylose units by the total number of reducing ends gives the average length of the polymers.

To determine the xylose content, the method of Coelho et al. [58] was adopted with modifications. Thus, 0.5 mg of sample was mixed with 5 mL H_2_SO_4_ 4% (*w*/*v*) and autoclaved at 121 °C and 1 bar pressure for 20 min to break down all the polymers into monomers. Samples were neutralized with 4M NaOH. After the centrifugation at 4000 rpm for 20 min, supernatant was filtered through 0.22 µm polyethersulfone filter (Merck, Darmstadt, Germany) for HPLC analysis. HPLC was performed in Shimadzu (Shimadzu Corp., Kyoto, Japan) chromatograph (LC-20AD pump, SIL-20A HT autosampler, CTO-10ASVP oven, DGU-20A5R degasser, CMB-20 A communication module) with Benson BP 800-Pb, 300 × 7.8 mm × 25 µm column (Benson Polymeric Inc., Reno, NV, USA) at 50 °C using RID detector with 5 mM H_2_SO_4_ mobile phase at a rate of 0.1 mL/min.

To determine the concentration of the reducing end terminals, 0.25 mL sample was added to 0.5 mL DNSA solvent and mixed at 100 °C for 10 min and cooled on ice for 5 min. Absorbance was measured with a spectrophotometer at 546 nm three times per sample. The calibration curve was created with 0.25 mL of different xylose concentrations treated with the same procedure as the samples. Average degree of polymerization (avDP) was calculated as follows:

(5) $$\mathrm{avDP}=\frac{\mathrm{Total}\;\mathrm{xylose}\;\mathrm{concentration}\;\mathrm{after}\;\mathrm{complete}\;\mathrm{hydrolysis}}{\mathrm{Concentration}\;\mathrm{of}\;\mathrm{reducing}\;\mathrm{ends}}$$

Xylan contents of the extracts were calculated from the determined xylose concentration using an anhydro correction factor as follows [59]:

(6) Xylan (mg)=Xylose (mg)×0.88

#### 3.3.5. NMR

NMR (nuclear magnetic resonance) analysis was performed using VNMRS 500 MHz spectrometer (Varian Inc., Palo Alto, CA, USA) with VnmrJ software (version 4.2, revision A; Agilent Technologies, Santa Clara, CA, USA). The lyophilized samples (50 mg) were dissolved in deuterated dimethyl sulfoxide (DMSO-*d*_6_) and transferred to NMR tubes. ^1^H NMR spectra were recorded at 25 °C. Each spectrum was acquired with 128 scans [57].

## 4. Conclusions

We examined the microwave effect on xylan extraction from corncob with two different alkali solutions. Through RSM analysis, the optimum conditions within the space of design were found to be 1200 W of microwave power for 20 min with 10% of alkali solution, which are the upper limits of the factors. Extracts of KOH and NaOH contained 77.82% and 72.01% xylan, respectively. Even though a significant portion of the xylan was extracted in a significantly shorter time, compared to the literature, further investigation with higher values of conditions should be conducted to determine the absolute optimum conditions, especially in terms of microwave power and alkaline solution concentration.

## Figures and Tables

**Table 1:** Microwave-assisted extraction results.

Sample	Microwave Power (Watt)	Time (min)	Alkali Concentration % (*w*/*v*)	Extract Yield of K-X * % (*w*/*w*)	Extract Yield of Na-X ** % (*w*/*w*)
1	800	11	5.5	19.036	24.732
2	800	11	5.5	13.616	25.58
3	400	20	5.5	18.064	21.352
4	1200	11	1	1.764	9.588
5	1200	20	5.5	24.796	36.556
6	1200	2	5.5	16.884	21.44
7	800	20	10	27.348	43.74
8	1200	11	10	32.116	27.152
9	800	11	5.5	13.232	26.112
10	800	20	1	1.556	6.812
11	400	11	10	2.6536	25.032
12	400	2	5.5	8.788	15.596
13	800	2	10	26.38	18.556
14	400	11	1	0.82	1.556
15	800	2	1	0.488	6.492

* Extractions with KOH. ** Extractions with NaOH.

**Table 2:** Analysis of variance (ANOVA) results of K-X (extraction with KOH) and Na-X (extraction with NaOH).

Source	Adj. Sum of Squares		DF		Adj. Mean Square		F-Value		*p*-Value	
	KOH	NaOH	KOH	NaOH	KOH	NaOH	KOH	NaOH	KOH	NaOH
Model	1338.36	1747.25	3	5	446.120	349.450	16.08	38.80	0.000	0.000
A-Microwave Power	18.78	121.66	1	1	18.782	121.664	0.68	13.51	0.428	0.005
B-time	NS	6.50	NS	1	NS	6.502	NS	0.72	NS	0.418
C-concentration	12.67	211.10	1	1	12.673	211.095	0.46	23.44	0.513	0.001
AC	203.33	NS	1	1	203.333	NS	7.33	NS	0.020	NS
BC	NS	154.55	NS	1	NS	154.555	NS	17.16	NS	0.003
C^2^	NS	189.01	NS	1	NS	189.014	NS	20.98	NS	0.001
Error	305.12	81.06	5	5	27.739	9.007				
Lack of Fit	284.05	80.10	9	7	31.562	11.442	3	23.62	0.275	0.041
Pure Error	21.07	0.97	2	2	10.53	0.484				
Total	1643.48	1828.31	14	14						

**Table 3:** Fit of statistics of K-X (extraction with KOH) and Na-X (extraction with NaOH).

Solution	Std. Dev.	R^2^	Adjusted R^2^
KOH	5.2	0.814	0.764
NaOH	3	0.956	0.931

**Table 4:** Zeta potential values of the xylan standard and the extracts.

	Xylan Standard	K-X (Extraction with KOH)	Na-X (Extraction with NaOH)
	−5.82	−4.41	−3.25
	−9.14	−4.28	−4.11
	−9.51	−4.14	−3.98
Average	−8.16 ± 2.03 ^a^	−4.28 ± 0.14 ^b^	−3.78 ± 0.46 ^b^

a, b: different letters within a row indicate significant differences at *p* ≤ 0.05 according to ANOVA.

**Table 5:** Xylan content (%) and degree of polymerization of the extracts.

Sample	Xylan (%)	avDP
Xylan Standard	100 ± 0.14 ^a^	22.69 ± 0.27 ^a^
K-X (extraction with KOH)	77.12 ± 1.61 ^b^	9.1 ± 0.31 ^b^
Na-X (extraction with NaOH)	73.52 ± 2.37 ^b^	10.42 ± 0.74 ^b^

a, b: different letters within a column indicate significant differences at *p* ≤ 0.05 according to ANOVA.

**Table 6:** Determination of variables.

Variables	Code	Range and Level
Microwave power (Watt)	A	400–1200
Time (min)	B	2–20
Alkali Concentration % (*w*/*v*)	C	1–10

**Table 7:** RSM experimental design.

Sample	Microwave Power (watt)	Time (min)	Alkali Concentration % (*w*/*v*)
1	800	11	5.5
2	800	11	5.5
3	400	20	5.5
4	1200	11	1
5	1200	20	5.5
6	1200	2	5.5
7	800	20	10
8	1200	11	10
9	800	11	5.5
10	800	20	1
11	400	11	10
12	400	2	5.5
13	800	2	10
14	400	11	1
15	800	2	1

## Data Availability

The original contributions presented in the study are included in the article; further inquiries can be directed to the corresponding authors.

## Abbreviations

The following abbreviations are used in this manuscript:

RSM	Response Surface Methodology
FT-IR	Fourier-Transform Infrared Spectroscopy
DSC	Differential Scanning Calorimetry
HMF	Hydroxymethylfurfural
Na-X	Xylan extractions with NaOH
K-X	Xylan extractions with KOH
DMSO-*d*_6_	deuterated dimethyl sulfoxide
HPLC	High-Performance Liquid Chromatography
NMR	Nuclear Magnetic Resonance
ANOVA	Analysis of variance
